# Treatment of the Throat, Nose and Ear in Scarlet Fever

**Published:** 1897-01

**Authors:** Henry Jackson

**Affiliations:** Assistant Visiting Physician of the Boston City Hospital, Boston; 309 Marlborough Street


### The Treatment of the Throat, Nose and Ear in Scarlet Fever.

BY HENRY JACKSON, M. D.,
Assistant Visiting Physician of the Boston City Hospital, Boston.

In determining the proper treatment of the throat and nose in scarlet fever, we must first consider the various pathological processes which are found in the naso-pharynx, either as characteristic lesions of the disease, or as severe and not unusual complications. First, practically in all cases, the pillars of the pharynx, uvula and often the posterior pharyngeal wall are the seat of a bright scarlet macular eruption, similar to the eruption on the surface of the body; this eruption precedes the skin lesion by twelve or twenty-four hours, and is the cause of the "sore throat" complained of by the patient with the onset of the fever. In mild cases, and in fact, in the majority of cases, within a few days there is but little complaint on the part of the patient of pain in the throat. In a few severe cases this erythematous process may be much more marked, and the swelling be such as to give rise to much pain in swallowing, and be a cause of great discomfort.

Second, we have pseudo-membranous lesions of the palate: this process may spread to the posterior pharyngeal wall and hence, after invasion of the vault of the pharynx, to the nose. This condition is usually found on the third or fourth day of the disease, and is the direct cause of the most alarming and fatal complications of scarlet fever; namely, septicæmia, otitis media purulenta, and infection of the cervical glands. Further, it is highly probable that serious kidney lesions found in cases of this class are the direst result of the sepsis rather than dependent upon the specific etiological factor of the scarlet fever. In rare cases we find a truly gangrenous condition, with destruction of tissue. Bacteriologically we find streptococci in the pseudo-membranous processes, organisms, which cannot be distinguished microscopially or biologically from the streptococcus pyogenes found in various severe septic processes and in erysiselas.

Third, there may be associated with the scarlet fever, diphtheria, giving rise to the growth of a false membrane which cannot clinically be distinguished from the process just described.

In cases of the first class we may relieve the discomfort by allowing the patient to frequently take small bits of ice into the mouth, and by irrigating the mouth with hot saline solutions. Further, as we do not know what is the cause of the serious pseudo-membranous lesion, we should from the first make use of non-irritating antiseptic washes. For this purpose, let the mouth be thoroughly irrigated several times a day with a hot four per cent. solution of boric acid. If the pain is severe, it may be relieved by using the following modified Dobell's solution in a hand atomizer:

| | |
|---|---|
| Sodii bicarb | 1.0 |
| Sodii chloridi | 1.0 |
| Sodii biborat | 2.0 |
| Glycerini | 25.0 |
| Aq. Rosæ, ad | 100.0 |

If necessary, small and frequently repeated doses of Dovers' power may be given.

Chlorate of potash, on account of its possible irritating effect on the kidney, is contraindicated.

The nose should be kept as clean as possible; to do this clean the anterior nares thoroughly with pledgets of absorbent cotton moistened with a four per cent. solution of boric acid. If necessary, the nose may be sprayed with the same solution, care being taken that the child does not snuff up the solution while it is being sprayed. I consider irrigation of the nose as inadvisable. This extreme caution is necessary in order to avoid inducing an inflammation of the Eustachian tubes, and a consequent otitis media. It was the experience of the aurist at the City Hospital that when douches were employed as a routine treatment, that disease of the ear was much more common than when the nose was not so treated. In a simple, uncomplicated case of scarlet fever the nose is not affected.

If pseudo-membranous deposit is found in the throat, a culture should be made at once, especially in hospitals where cases of scarlet fever and diphtheria are accepted; if the Klebs-Loeffler bacilli of diphtheria are found, the child should be given the benefit of the antitoxine diphtheria.

If false membrane is found in the throat, the utmost care should be taken to prevent, if possible, the spreading of the membrane. In scarlet fever the danger of extension of the membrane to the larynx is very small; on the other hand, the membrane is apt to spread to the naso-pharynx and nose, giving rise to a serious septicæmia, which is the most fatal complication of scarlet fever. Furthermore, the glands of the neck are often involved, and there is great danger of a purulent otitis media, due to an extension of the septic process through the Eustachian tube. The throat should be thoroughly irrigated every two hours with a hot, saturated solution of boric acid; or a solution of liquor sodii chlorinati may be used in the strength of one part to sixteen parts of water, this solution having the advantage of being a good deodorizer. A twenty-five volume solution of peroxide of hydrogen may be used with great advantage; it is to be applied in a spray apparatus;(using an apparatus made entirely of glass, as devised by Dr. Williams), several times a day, and followed by irrigation.

If the nose is involved in the growth of false membrane, it is neccessary to cleanse it. Now the danger of causing ear disease is much greater, and the nose must be most carefully cleasned with pledgets of cotton before it is sprayed; the nose should then be carefully sprayed with four per cent. boric acid solution, and when the nostrils are free, the same solution should be poured into the nose, taking care not to use pressure, as given by a douche bag placed at a height above the patient. The discharge from the nose and mouth is very irritating to the skin, therefore the lips and the alæ nasi should be carefully wiped, and then smeared with vaseline.

It remains for the future to decide whether we are to find in anti-streptococcus serum a specific for the various septic processes produced by the streptococci. Marmorek is enthusiastic over the results which he has obtained in treating by this method the serious throat lesions and the enlarged cervical glands of scarlet fever.

A rare and serious complication of scarlet fever is a post-pharyngeal abscess. If such an abscess is formed, it must be opened. Great care is necessary, if the patient is etherized, to prevent the pus from entering the trachea. I have seen a case in which the life of the patient was saved only by an immediate tracheotomy.

If the ear is involved, we have as evidence thereof severe earache; this may at first be treated by hot applications behind the ear, and small doses of Dover's powder. If the pain does not subside in twenty-four hours, an aurist should be called, as an immediate incision of the drum with inflation may in many cases save the patient from weeks of suffering as the result of purulent otitis. If there is a discharge from the ear, small pledgets, "wicks," of absorbent cotton should be placed as far into the meatus as possible, and changed as often as they are wet. If tenderness develops over the mastoid process, and the surgeon finds redness with slight œdema, he must be ready at any moment to trephine the mastoid process in order to prevent a possible inflammation of the brain from extension of the process inwards.—*Archives Pediatrics.*

309 Marlborough Street.

